# Prevalence of Successful Aging in the Elderly in Western Mexico

**DOI:** 10.1155/2012/460249

**Published:** 2012-09-25

**Authors:** Elva Dolores Arias-Merino, Neyda Ma. Mendoza-Ruvalcaba, Martha Judith Arias-Merino, Jazmín Cueva-Contreras, Carlos Vazquez Arias

**Affiliations:** University Center for Health Sciences, University of Guadalajara (UDG), 44348 Guadalajara, JAL, Mexico

## Abstract

*Objectives*. The aim of this paper is to estimate the prevalence of successful aging in the elderly in Western Mexico and to analyze its variability by age, sex, education, marital status, and pension. *Methods*. This study employs data from the Health, Wellbeing, and Aging Study (SABE) in Jalisco and Colima, Mexico. Successful aging was operationalized in accordance with no important disease, no disability, physical functioning, cognitive functioning, and being actively. There were a total of 3116 elderly. *Results*. 12.6% of older adults were “successful” aging. The old-old is a lower proportion of successful aging people; it ranges from 18.9% among people aged 60–69 years to 3.9% in the 80–89 years and up to 1% in people 90 and older. There were also differences according to sex (*P* = .000), with a higher proportion of successful aging men (18.4% compared with 9.2% of women). There were differences in educational level (*P* = .000); those higher with education were found to be more successful aging, and also there were differences in marital status for married people (*P* = .000). *Discussion*. A small number of older adults meet the criteria definition of successful aging, suggesting the need to analyze in depth the concept and the indicators.

## 1. Introduction

The increase in the relative and absolute number of older people in our society has posed a major challenge for both individual and collective levels in the study of aging. It had appeared various contributions, at the individual level, extending the biological perspective of disease and disability with a more positive and comprehensive one, covering the social and behavioral sciences to reduce the risk of adverse events and improve the resilience of the seniors and make changes in their immediate surroundings [1]. 

At the population level, aging is seen as a challenge that involves, in addition, as stated by Fernandez-Ballesteros [2], challenges such as the double burden of disease, increased risk of disability, having to provide adequate care for the aging population, addressing inequalities, economic challenges, and having a different view of aging and old age.

In industrialized countries, demographic and social changes of the population have brought into discussion the approach of public policies on aging related to pensions, employment, health social care, and protection of citizenship. In this context emerges a new paradigm that implies a new vision of aging, a positive vision, called “active aging”. *The WHO has considered that Active Ageing is the key if it is wanted to make aging a positive experience and free of disability, with ongoing opportunities for health, participation and security especially in increasingly aging societies like ours* [3]. 

The concept of active aging emphasizes the vital connection between activity and health since it considers active aging in terms of health, independence, and productivity of older people. It also incorporates key principles to meet the policy domains required to successfully meet the challenges of an aging population: the activity, prevention, inclusion of all older people, maintenance of intergenerational solidarity, rights and obligations, participation and empowerment of the people, and respect for national and cultural diversity (for review, see Alan Walker [4, 5]). 

Alan Walker [5] performed an important review of the theoretical foundations of the term active aging and explains that the use of it is much older in the United States, dating back to the 1960s, [6] initially taking the name of “successful aging” is to keep in the old age the same activity patterns and values typical of the middle age.

Regarding this issue, Rowe and Kahn [1, 7, 8] made an important contribution, with a theoretical model of “successful aging” at the individual level covering three different areas: preventing disease and disability, maintaining high physical and cognitive function, and to having a sustained commitment to social and productive activities. This model has been widely used as a tool to describe the aging of the elderly; [9–21] however, as aging is a dynamic process it may vary over time by the influence of the social, economic, and political dimensions [22, 23].

In this study we will use the model of Rowe and Kahn on successful aging considering that active aging encompasses both macro- and microstructural dimensions and that, occurs over time as active aging leads to successful aging.

This model considers that there are different forms of aging: usual, pathological, and successful. Fernandez-Ballesteros [24] mentions that successful aging can be considered as a categorical variable that can estimate prevalence in the population.

The aim of this paper is to estimate the prevalence of successful aging in the elderly in western Mexico as defined by Rowe and Kahn and analyze its variability by age, sex, education, marital status and pension.

## 2. Methods

### 2.1. Population and Procedures

This study employs data from the Health, Wellbeing, and Aging Study (SABE) in Jalisco and Colima, Mexico. It is a cross-sectional study proposed for Latin American population by the PAHO; it consists in a protocol to assess health, functionality, nutritional, cognitive, emotional, and social aspects of elderly [25]. A total of 3,116 elderly persons were included in a multistage, proportional, and randomized sample that included the states of Jalisco (*n* = 1596) and Colima (*n* = 1520) (in western Mexico). Maps and databases of potential populations were consulted. To carry the survey out in the geographical area, study areas were defined through the National Geo-statistic Framework (INEGI). Basic Geo-statistical Areas (*AGEBs*) were chosen randomly, and the same was done for regarding blocks and homes until finding the study subjects. Persons 60 years and older were invited to participate in the study. Characteristics of the study participants are shown in Table 1; 15.8% of people had cognitive impairment, which was requested an informed as suitable to respond to the interview. Ethical approvals in both states and informed consent or relative agreement were obtained.

### 2.2. Dependent Variable

Based on the conceptualization of Rowe and Kahn [1] of successful aging and studies by Strawbridge et al. [19] and McLaughlin et al. [22] successful aging is defined as having (a) no important disease, (b) no disability in activities of daily living (ADLs), (c) no more than one difficulty of seven measures of physical functioning, (d) cognitive functioning, and (e) being actively engaged. (for review, see McLaughlin et al. [22], SABE study included same indicators in the survey).

**No Important Disease**

Participants in SABE study were asked that if ever a doctor or nurse has told them to have each of the following five chronic diseases: cancer, chronic lung disease, diabetes, heart disease and stroke (for this analysis hypertension and articulate disease were excluded). We have also included a measure of mental health as McLaughlin refers [22], in the SABE study, we applied the Geriatric Depressive Screening scale (GDS) with scores ranging from 0–15. We considered subjects with high depressive symptoms those with a score >5. To meet the criteria of “lack of serious illness”, respondents could not have any of the five chronic diseases and depressive symptoms as classified [26].

**No Disability**

Respondents who reported no difficulty performing each of the six basic activities of daily living ADLs (i.e., walking across a room, dressing, bathing or showering, eating, getting in or out of bed, and toileting) met the criterion of no disability [12, 19].

**Physical Functioning**

Participants were classified as with high physical functioning if did not reported more than one difficulty with any of the following seven measures, including walking one block, walking several blocks, climbing up one floor of stairs, climbing several floors of stairs, lifting or carrying the items weighing more than 10 pounds, stooping, kneeling, stooping or squatting, and pulling or pushing big objects. The SABE survey measures are identical to the study of McLaughlin et al. [22] which has its background in Seeman et al. [13].

**Cognitive Functioning**

Cognitive impairment was measured using the Mini-Mental State Examination of the SABE protocol [27]. The MMSE score was calculated using the sum of correct answers (0–19 points), the cutoff point was 12/13.

**Being Actively Engaged**

It refers to social connections and participation in productive activities [1, 8]. For this analysis, it is defined as “actively participate” if the participant reported doing any paid work during the last week from the interview, or any volunteer work, family, home or selling on their own. In addition to reporting any of these social connections: being married or living in company with a relative or friend, and if participants attend religious celebrations often.

### 2.3. Independent Variables

Age, gender, education, marital status, and pension were included in the analysis. Age was categorized as 60–74 years and 75 and older. Education was categorized as less than high school and secondary or higher education level.

### 2.4. Analytic Techniques

The prevalence of successful aging was calculated by age, gender, education, marital status, and pension. To determine if sociodemographic differences were statistically significant, the adjusted odds ratio (OR) and 95% confidence intervals (CIs) were calculated. The independent associations between age, sex, education, marital status and pension, and successful aging were evaluated by binary logistic regression analysis (confidence intervals by exp *β* of 95%).

## 3. Results

Table 1 shows the sociodemographic characteristics of participants. The mean age was 72.41 (SD = 8.47) years, the majority of participants were women (62.5%). Regarding education, 20.4% were illiterate, 57.0% had less than secondary education, and only 22.6% more than high school. Most of the elderly were married (55.5%) and 6.4% never did so. Only 21.2% had pension.

While comparing the sociodemographic characteristics by gender we see that 45.6% of women were between 60 and 69 years old as opposed to 37.8% of men, this difference affects the average age where women obtained 72.0 ± 8.54 and 73.0 men ± 8.33 years.

At the same time, men mentioned higher levels of education than women, for example, 26.8% of men have high school or higher level of education, while on the other hand only 20.0% of women have it. Regarding to marital status, 71.5% of men claimed to be married or cohabiting, while only 45.9% of women did. These gender differences are more marked on the pension, as only 9.4% of women report receiving pension compared with 41.0% of men.

The ratio of participants meeting the criteria of successfully aging was calculated, the results are shown in Table 2. As it can be seen, a higher percentage met the criteria of cognitive functioning (84.2%), men (86.4%) in greater proportion than women (83.0%), and no disability (74.2%), while 41.0% and 39.3% met the criteria of nondisease and physical functioning, respectively. The latter with significant differences by sex (33.0% women and 49.8% men).

 The criterion of social commitment was met by 48.5% of the participants; women also had lower percentage (43.2%) than men (57.3%).

Altogether, 12.6%  of the participants met all the criteria to be considered as successfully aging. When comparing among successfully aged elderly (see Table 3) significant differences (*P* = .000) according to age were found. In older age there is a lower proportion of active aging people, it ranges from 18.9% among those between 60–69 years to 3.9% among those between 80–89 years and up to 1% in people 90 and older. There were also differences according to gender (*P* = .000), with a higher proportion of men aged successfully (18.4% compared to 9.2% of women). In the same way, differences in educational level (*P* = .000), whereas education level increasing more active aging was found, and also differences in marital status for married people (*P* = .000). There were no differences according to whether receiving pension or not (*P* = .093).

Finally crude and adjusted odds ratios and confidence intervals for successful aging were calculated, the results are shown in Table 4.

As seen in the unadjusted analyses, 30.0% of adults of 75 or more years had the possibility of successful aging compared to the group of 60 to 74 years, OR = 0.30 (0.23 to 0.39, CI 95%). Women had 50.0% less successful aging than the observed in men, OR = 0.50 (0.41 to 0.60, CI 95%). Participants with lower education had only 55.0% successful aging compared to those with higher education, OR = 0.55 (0.45 to 0.66, CI 95%).

Finally, the elderly that at the time of the interview were not married or cohabiting had only 18% of successful aging compared to those who were married, OR = 0.18 (0.13 to 0.24, CI 95%). After adjusting for sociodemographic factors (age, sex, education and marital status) these four are held as factors in the model of successful aging. The adjusted odds ratios are slightly higher than the unadjusted.

## 4. Discussion

In this study the prevalence of successful aging was 12.6%. Mexico is currently in the process of demographic transition towards an aging population, even though the life expectancy is lower than that in developed countries. It is not known whether in Mexico there are other studies that estimate the prevalence of successful aging or not, so the comparison of our results with studies from developed countries is flat and should be viewed with caution, given the differences in the age structure of aging population and the social and economic conditions.

Other active aging studies that were based on the criteria of Rowe and Kahn found 18.8% [19] and 11.9% to 10.9% [22] of prevalence of successful aging. Several review studies have found a large variability in the indicators that have an influence on how the prevalence of successful aging is defined, having a direct effect on the reported figures. For example, Depp and Jeste [28] reviewed 27 studies where besides defining the concept operationally, they established the prevalence of successful aging and found a range that varied from 0.4 to 95%. In a similar review study by Peel et al. [29] 18 studies that included definitions of successful aging were analyzed and it was found that the prevalence established in these studies ranged from 3% to 80%. With this, it can be established that the nature of definitions, domains, and selected measures results in considerable variation in the proportion of the population classified as successful aging. Overall, we had a low nonparticipation rate estimated in 8.67%. The population study consisted of a greater number of women (62.5%) than men (37.5%) with an average age less than men. The reason could be because the interviews were conducted in homes, and men, particularly the younger, still have a working life.

Regarding gender, in this study it was found that more men than women meet the criteria of Rowe and Kahn's successful aging. This is a controversial finding, since other studies have found opposite trends where more women than men have higher prevalence of successful aging. For example Strawbridge et al. [19] reported significant differences when finding that 21.5% of women and 15.4% of men met the criteria for successful aging, while McLaughlin et al. [22] found a trend towards men but not significant one. However, we think that in our female population the prevalence is consistent with the data, as these are seen as linked to lower cognitive and physical function as well as less social and productive participation. Also, it is observed in women a lower level of education, a significant number of widows, and almost no pension (9.4%).

The results regarding the relation between age and level of education with successful aging are more consistent in the sense that as age increases, the percentage of people with successful aging decreases, while higher education prevalence of successful aging among the population increases significantly.

A limitation of this study is that some of the criteria for successful aging are based on self-referrals of both health status and physical activity. However, the criterion of cognitive function was actually evaluated and in some way helps to ensure that the classification is not based only on subjective judgments. In the same way, the assessment of depressive symptoms was performed using a validated scale.

It is also necessary to consider that 15.8% of the sample had cognitive impairment and was asked to perform a key informant interview. Thus, this study found 84.2% of cognitive function, similar to the numbers found in a study of prevalence of cognitive impairment in Jalisco population, estimated in 85.5% [30].

Regarding no disability estimated in 74.2%, it was found in our population a lower percentage of no disability than the other seven populations of Latin American cities, in the SABE study, who ranged from 76.3%–86.2% of no disability [31].

Another limitation is that our study is about prevalence so that the associations are horizontal and do not allow the establishment of predictors. However, this is a first approach that contributes to the characterization of the Mexican population around the concept of successful aging.

## 5. Conclusions

This study presents a research on the prevalence of successful aging in elderly in western Mexico. A small number of elderly met the criteria of Rowe and Kahn definition of successful aging, so more effort is required in the individual and collective levels to improve the health, economic, and social participation conditions, as well as greater efforts to establish public policies in accordance with the principles of active aging in different dimensions from the macro-, meso- and microstructural that include people of all ages, so that different generations can age actively and achieve successful aging.

Regarding the criteria for successful aging, Rowe and Kahn proposal includes biological, psychological, and social aspects, which gives the desired multidimensionality at an individual level to define successful aging. However, it is in the operationalization and the establishment of indicators where more efforts should be done to reach consensus and achieve comparative studies to reach with it more consistency.

## Figures and Tables

**Table 1:** Socio-demographic Characteristic by Sex.

Characteristic	Women % (*n*)	Men % (*n*)	Total % (*n*)
Sex***	62.5 (1949)	37.5 (1167)	100.0 (3116)
Age, years***			
60–69	45.6 (889)	37.8 (441)	42.7 (1330)
70–79	34.0 (662)	39.4 (460)	36.0 (1122)
80–89	17.4 (340)	19.2 (224)	18.1 (564)
90+	3.0 (58)	3.6 (42)	3.2 (100)
Education (level)***			
Did not attend school	21.0 (409)	19.5 (228)	20.4 (637)
Less than high school	59.0 (1150)	53.6 (626)	57.0 (1776)
High school or higher	20.0 (390)	26.8 (313)	22.6 (703)
Marital status***			
Married	45.9 (894)	71.5 (834)	55.5 (1728)
Widowed/separated/ divorced	46.4 (904)	24.5 (286)	38.2 (1190)
Never married	7.7 (151)	4.0 (47)	6.4 (198)
Pension***			
Yes	9.4 (184)	41.0 (478)	21.2 (662)
No	90.6 (1785)	49.0 (689)	78.8 (2454)

***P = .000.

**Table 2:** Percentage of older adults meeting each individual successful aging criterion and total percentage.

Criteria	Women % (*n*)	Men % (*n*)	Total % (*n*)
No major disease	40.1 (761)	42.5 (484)	41.0 (1245)
No disability	73.1 (1414)	76.1 (884)	74.2 (2298)
Cognitive functioning*	83.0 (1617)	86.4 (1008)	84.2 (2625)
Physical functioning***	33.0 (643)	49.8 (581)	39.3 (1224)
Social engagement***	43.2 (842)	57.3 (669)	48.5 (1511)

Successful aging***	9.2 (179)	18.4 (215)	12.6 (394)

*P = .01  ***P = .000.

**Table 3:** Successful aging by age, sex, education, marital status and pension.

Variable	Successful aging % (*n*)	No Successful aging % (*n*)	*P* = *
Age (years)			
60–69	18.9 (251)	81.1 (1079)	.000
70–79	10.7 (120)	89.3 (1002)	
80–89	3.9 (22)	96.1 (542)	
90+	1.0 (1)	99.0 (99)	
Sex			
Women	9.2 (179)	90.8 (1770)	.000
Men	18.4 (215)	81.6 (952)	
Education (level)			
Did not attend school	5.8 (37)	94.2 (600)	.000
Less than high school	12.4 (220)	87.6 (1556)	
High school or higher	19.5 (137)	80.5 (566)	
Marital status			
Married	19.9 (344)	80.1 (1384)	.000
Widowed/separated/ divorced	2.9 (35)	97.1 (1155)	
Never married	7.6 (15)	92.4 (183)	
Pension			
Yes	13.9 (92)	86.1 (570)	.093
No	12.3 (302)	87.7 (2152)	

*Chi-square test.

**Table 4:** Crude and adjusted odds ratio for successful aging.

Variable	Successful aging (%)	Crude OR (95% CI)	*P* =	Adjusted OR* (95% CI)	*P* =
Age, years					
≥75	5.2	0.30 (0.23–0.39)	.000	0.35 (0.26–0.47)	.000
60–74	17.0				
Sex					
Women	9.2	0.50 (0.41–0.60)	.000	0.52 (0.41–0.66)	.000
Men	18.4				
Education					
≤high school	10.7	0.55 (0.45–0.66)	.000	0.58 (0.45–0.75)	.000
>high school	19.5				
Married status					
No	3.6	0.18 (0.13–0.24)	.000	0.20 (0.14–0.27)	.000
Yes	19.9				

Notes: adjusted for age, sex, education and marital status.

OR: odds ratio, CI: confidence interval, *binary logistic regression.
